# Highly efficient PCR assay to discriminate allelic DNA methylation status using whole genome amplification

**DOI:** 10.1186/1756-0500-4-179

**Published:** 2011-06-10

**Authors:** Yoichi Yamada, Takashi Ito

**Affiliations:** 1 School of Electrical and Computer Engineering, College of Science and Engineering, Kanazawa University, Kanazawa 920-1192, Japan; 2 Department of Computational Biology, Graduate School of Frontier Sciences, University of Tokyo, Kashiwa 277-8561, Japan

## Abstract

**Background:**

We previously developed a simple method termed *Hpa*II-*McrBC *PCR (HM-PCR) to discriminate allelic methylation status of the genomic sites of interest, and successfully applied it to a comprehensive analysis of CpG islands (CGIs) on human chromosome 21q. However, HM-PCR requires 200 ng of genomic DNA to examine one target site, thereby precluding its application to such samples that are limited in quantity.

**Findings:**

We developed *Hpa*II-*McrBC *whole-genome-amplification PCR (HM-WGA-PCR) that uses whole-genome-amplified DNA as the template. HM-WGA-PCR uses only 1/100th the genomic template material required for HM-PCR. Indeed, we successfully analyzed 147 CGIs by HM-WGA-PCR using only ~300 ng of DNA, whereas previous HM-PCR study had required ~30 μg. Furthermore, we confirmed that allelic methylation status revealed by HM-WGA-PCR is identical to that by HM-PCR in every case of the 147 CGIs tested, proving high consistency between the two methods.

**Conclusions:**

HM-WGA-PCR would serve as a reliable alternative to HM-PCR in the analysis of allelic methylation status when the quantity of DNA available is limited.

## Findings

### Background

The Human Genome Project has contributed to progress in various research fields including epigenetics (i.e., the study of the phenomena that regulate gene expression without alteration of genomic sequences). Various posttranslational modifications of histones and DNA methylation represent typical epigenetic events [1]. Most CpG dinucleotides in the mammalian genome are modified by a methyl group at the C5-position of the cytosine. CpG dinucleotides occur in mammalian genomes less frequently than would be expected from the GC-content of DNA, because methylated CpG frequently converts to TpG, while unmethylated CpG does not [2]. However, there are CpG-rich regions, called CpG islands (CGIs), in the mammalian genome [3]. It is thought that CGIs are able to keep their CpG-rich sequences, because they generally escape methylation [4]. CGIs often lie in the promoter regions of house-keeping genes and are occasionally found associated with tissue-specific genes [3]. However, CGIs on the × chromosomes of females and those around imprinted genes are subject to monoallelic methylation in a random and a parent of origin-dependent manner, respectively [5]. Aberrant methylation is also frequently observed in various types of cancer cell [6].

DNA methylation contributes to the regulation of gene expression, as well as to the suppression of parasitic sequences, inactivation of the × chromosome in females, genomic imprinting, and maintenance of chromosome stability. Thus, it is important to profile DNA methylation at human gene promoters in each type of adult tissue and during different stages of human development [7]. For this purpose, we previously developed a method called *Hpa*II-*McrBC *PCR (HM-PCR) that fully exploits reference sequence data to reveal allelic methylation status of genomic sites of interest [8]. It has been successfully applied to comprehensive analyses of allelic methylation status of CGIs on human chromosomes 21q [8] and 11q [9] in peripheral blood cells as well as 181 alternative promoters of 61 human genes in five different tissues [10]. Our analysis of chromosome 21q not only provided one of the first lines of evidence for CGI methylation in normal tissues, but reported a first case of nonimprinted, sequence-dependent allele-specific methylation (ASM) [8]. The latter finding was followed by a series of recent studies that uncover a wide occurrence of SNP/haplotype-dependent ASM often associated with allele-specific expression of nearby genes [11,12]. These findings underscore the importance of analyzing allelic methylation status to reveal both imprinted and nonimprinted forms of ASM.

In this context, it should be noted that HM-PCR serves as a much simpler assay for ASM than any other ones including bisulfite conversion. It, however, requires 200 ng of genomic DNA to examine the methylation status of one target site. Accordingly, HM-PCR is not suitable for a comprehensive analysis of allelic methylation status when the quantity of sample DNA is limited.

To overcome this problem, we integrated a method for whole genome amplification (WGA) with HM-PCR to develop HM-WGA-PCR. This method allows us to examine allelic methylation status using only 1/100th the amount of DNA required for the original HM-PCR with comparable accuracy.

## Methods

### Preparation of genomic DNA

Normal human genomic DNA from peripheral blood cells was obtained from Novagen. To purify genomic DNA, the DNA was incubated in 1 M NaCl solution overnight at 37°C and subjected to phenol-chloroform extraction. The DNA was recovered by standard ethanol precipitation and dissolved in TE (10 mM Tris-HCl (pH 8.0), 1 mM EDTA). For this human genome research, we have received ethical approval from Kanazawa University Ethics Committee (approval number: 231).

### HM-WGA-PCR

Genomic DNA (500 ng) was digested with 50 U of *Hpa*II, *Hha*I, *Msp*I (TaKaRa), or *McrBC *(New England Biolabs) overnight at 37°C in 50 μl of the recommended buffers. The digested DNA was recovered by standard ethanol precipitation and dissolved in 10 μl of TE (10 mM Tris-HCl (pH 8.0), 1 mM EDTA). Note that the amount of DNA used for digestion is dependent on the number of amplicons to be tested. For smaller amount of DNA, use of appropriate carriers would be helpful to ensure efficient recovery.

The DNA digested with each enzyme was then amplified using GenomiPhi Amplification Kit (GE Healthcare). We routinely use 50 ng of digested DNA for each amplification. Briefly, 1 μl (50 ng) of digested DNA prepared as above was mixed with 9 μl of sample buffer and then denatured for 5 min at 96°C. Following cooling down to 4°C, the denatured DNA was mixed with 9 μl of reaction buffer and 1 μl of phi29 DNA polymerase, and then incubated for two days at 30°C. For inactivation of phi29 DNA polymerase, the amplified DNA solution was heated for 10 min at 65°C. The amplified DNA was purified by ethanol precipitation and dissolved in 100 μl of TE. Here, ~5 μg of whole-genome-amplified DNA was obtained from 50 ng of digested DNA.

We used 1 μl (50 ng) of each WGA product prepared as above for each PCR in 10 μl of the recommended buffers containing 2.5 U of Ex-Taq DNA polymerase (TaKaRa) and 2.5 pmols of each primer. Since each HM-PCR assay involves 4 PCR, HM-WGA-PCR uses 200 ng (= 50 ng × 4) of amplified DNA in total, which is equivalent to 2 ng of original genomic DNA. Thermal cycling parameters and primer sequences used in this study are shown in Additional file 1: Supplemental Table S1.

### Bisulfite sequence analysis

Bisulfite sequencing was performed as described [8]. Thermal cycling parameters and primer sequences are described in Additional file 1: Supplemental Table S1.

## Results and discussion

**Figure 1:**
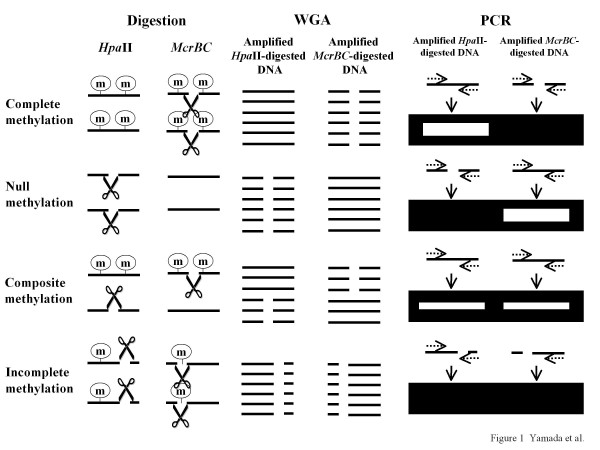
**Principles of the *Hpa*II-*McrBC *whole-genome-amplification PCR (HM-WGA-PCR) method**. The two parallel lines in the "Digestion" panel indicate genomic amplicons from both alleles. The circled "m" indicates a methylated CpG dinucleotide. *Hpa*II digests unmethylated CCGG, but not methylated C^m^CGG. In contrast, *McrBC *digests methylated R^m^C_40-80_R^m^C sequences, but not unmethylated RC_40-80_RC. Genomic DNA is digested with *Hpa*II and *McrBC *independently. Subsequently, an aliquot of each restriction-enzyme-digested DNA (50 ng) is subjected to whole-genome-amplification (WGA) to yield 5 μg of whole-genome-amplified DNA. Using an aliquot of the amplified DNA (50 ng), the target DNA region is PCR-amplified by the primer pair (dotted arrows). The PCR products from the *Hpa*II/*Hha*I-digested and *McrBC*-digested DNA are electrophoresed, stained with ethidium bromide, and visualized by UV illumination. If an amplicon is fully methylated (i.e., complete methylation), it is digested by *McrBC*, but not by *Hpa*II. Thus, it is amplified from the *Hpa*II-digested and whole-genome-amplified DNA, but not from the *McrBC*-digested and whole-genome-amplified DNA. By contrast, if an amplicon totally escapes methylation (i.e., null methylation), it is digested by *Hpa*II, but not by *McrBC*. Thus, it is amplified from *McrBC*-digested and whole-genome-amplified DNA, but not from *Hpa*II-digested and whole-genome-amplified DNA. If an amplicon contains both methylated and unmethylated alleles (i.e., composite methylation), it is amplified from both whole-genome-amplified templates. If an amplicon is partially methylated on both alleles (i.e., incomplete methylation), it is amplified from neither whole-genome-amplified template.

*Hpa*II-*McrBC *whole-genome-amplification PCR (HM-WGA-PCR) uses two types of restriction enzyme. One is either *Hpa*II (CCGG) or *Hha*I (GCGC), which digests unmethylated DNA, but not methylated DNA (i.e., methylation-sensitive enzymes). The other type is *McrBC *(R^m^CN_40-80_R^m^C), which cuts methylated DNA, but not unmethylated DNA (i.e., methylation-dependent enzyme) [13]. In HM-WGA-PCR, genomic DNA is first digested with either *Hpa*II/*Hha*I or *McrBC *and then subjected to whole-genome-amplification by phi29 DNA polymerase [14], which can uniformly amplify genomic DNA (Figure 1). Finally, PCR amplification of each amplicon is conducted by using the two whole-genome-amplified DNA samples as the templates (i.e., one from *Hpa*II/*Hha*I digestion and the other from *McrBC *digestion) (Figure 1).

Note that each amplicon is selected to contain both *Hpa*II/*Hha*I and *McrBC *sites. If an amplicon is biallelically methylated (i.e., complete methylation), it is amplified only from *Hpa*II-digested genomic DNA, but not from *McrBC*-digested DNA (Figure 1). Conversely, if an amplicon escapes methylation biallelically (i.e., null methylation), it is amplified only from *McrBC*-digested DNA, but not from *Hpa*II-digested DNA (Figure 1). If an amplicon is composed of both methylated and unmethylated alleles (i.e., composite methylation), it is amplified from both templates, because *Hpa*II/*Hha*I and *McrBC *fail to digest methylated and unmethylated alleles, respectively (Figure 1). If an amplicon is partially methylated on both alleles (i.e., incomplete methylation), it is amplified from neither template, because both enzymes digest both alleles (Figure 1). Accordingly, four different allelic methylation patterns (i.e., complete, null, composite, and incomplete methylation) can be clearly discriminated as four distinct amplification patterns (Figure 1).

HM-WGA-PCR is based on HM-PCR, which includes only two steps in Figure 1, namely "Digestion" and "PCR" [8]. A drawback of HM-PCR is that it requires 200 ng of genomic DNA to analyze methylation in one amplicon; 50 ng each for *Hpa*II-, *McrBC*-, *Msp*I- and mock-digestion, the latter two of which serve as controls. Therefore, it is not suitable for a comprehensive analysis of methylation when the quantity of available genomic DNA is limited. In HM-WGA-PCR, we input 500 ng of genomic DNA for each digestion and used 1/10th of the digested DNA (i.e., 50 ng) for WGA, which likely yielded ~5 μg of amplified DNA. We confirmed that 1/100th or 50 ng of the WGA product, which is equivalent to 0.5 ng of original genomic DNA, is enough for each PCR.

**Figure 2:**
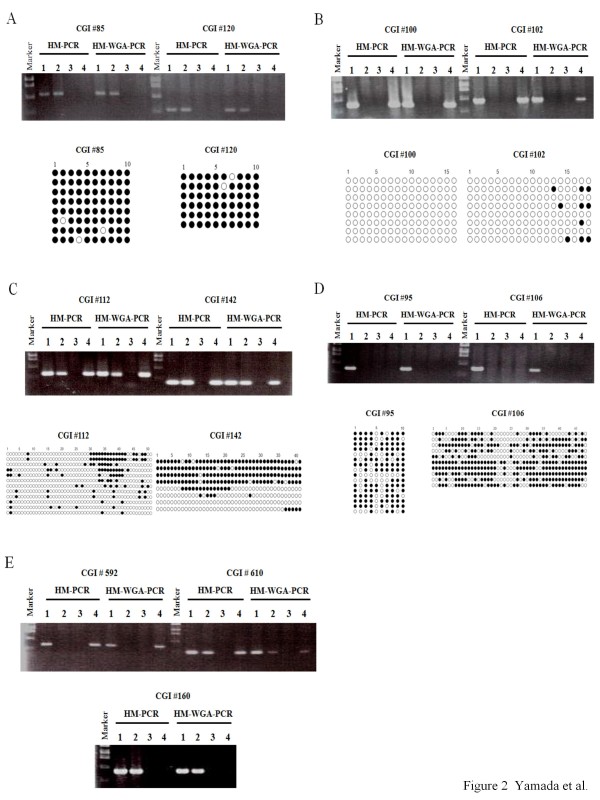
**Comparison of HM-PCR and HM-WGA-PCR results for eleven CGIs**. Genomic DNA from peripheral blood leukocytes was digested with Mock (lane 1), *Hpa*II or *Hha*I (lane 2), *Msp*I (lane 3), or *McrBC *(lane 4). The digested genomic DNA were used for PCR amplification either directly (left; HM-PCR) or after whole-genome-amplification (right; HM-WGA-PCR) using primer pairs for the eight CGIs on chromosome 21 (A~D) and three CGIs on chromosome 11 (E). Here, when *Hha*I-digested genomic DNA was used in lane 2, 1 ul of distilled water was used in lane 3 in place of *Msp*I-digested genomic DNA. PCR products were electrophoresed, stained with ethidium bromide, and visualized by UV illumination. Results of bisulfite sequencing are shown for the eight CGIs on chromosome 21 (A~D). Open and closed circles indicate unmethylated and methylated CpG dinucleotides, respectively. Each row of circles represents each sequenced clone of bisufite PCR products.

Since HM-WGA-PCR is basically identical to HM-PCR except for the WGA process, both methods should provide the same results on the same amplicon. We thus examined whether or not HM-WGA-PCR can distinguish between the four allelic methylation patterns as clearly as HM-PCR. For this purpose, we used primer pairs for eight CGIs on human chromosome 21q (#85, #95, #100, #102, #106, #112, #120, and #142) as representatives of the four allelic methylation patterns (Additional file 1: Supplemental Table S1). As shown in Figure 2A~2D, HM-PCR and HM-WGA-PCR provided the same patterns. In other words, both HM-PCR and HM-WGA-PCR determined that CGIs #85 and #120, #100 and #102, #112 and #142, #95 and #106, are modified by complete-, null-, composite-, and incomplete-methylation, respectively. These results suggested that HM-WGA-PCR can distinguish the four methylation status patterns on CGIs as distinctly as HM-PCR.

To test the robustness of HM-WGA-PCR more rigorously, we applied it to the 147 CGIs on human chromosome 21q that had been analyzed in our previous study (Additional file 1: Supplemental Table S1). While we previously analyzed 149 CGIs in total, we omitted CGIs #103 and #75 from this study for the following reasons. The former CGI lacks recognition sites for methylation-sensitive restriction enzymes and hence cannot be analyzed by HM-PCR; it had been analyzed by bisulfite sequencing in our previous study [8]. The latter CGI was omitted, because updated reference sequence data disproved the validity of the primers used for this CGI in the previous study (data not shown) [8].

We applied HM-WGA-PCR to the 147 CGIs and successfully amplified all of them, suggesting largely unbiased amplification by WGA. Furthermore, in every case of the 147 CGIs, allelic methylation status revealed by HM-WGA-PCR was exactly the same as that revealed by the HM-PCR; we thus achieved 100% consistency between the two methods (data not shown). Note that these HM-WGA-PCR assays consumed only 300 ng of genomic DNA, whereas our previous HM-PCR study of the same 147 CGIs had required 30 μg.

We applied HM-WGA-PCR to several CGIs on other chromosomes, including CGIs #160, #592, and #610 on chromosome 11q (Figure 2E), and confirmed that the results of HM-PCR and HM-WGA-PCR are consistent in all the cases examined (data not shown). We also confirmed that HM-WGA-PCR works well on placental DNA to give consistent results with our previous HM-PCR (data not shown).

Taken together, HM-WGA-PCR is much more efficient than HM-PCR and retains comparable accuracy to HM-PCR. It would thus provide a reliable alternative to HM-PCR, when examining allelic methylation status using a limited quantity of DNA.

Allelic methylation status can be also examined by methods based on bisulfite conversion of unmethylated cytosine (e.g., bisulfite sequencing, COBRA [15], and methylation-specific PCR [16]). While these methods as well as HM-WGA-PCR require a few ng of DNA for each assay and are thus comparable in terms of the amount of DNA required, each of them has its own advantages and drawbacks. For instance, bisulfite sequencing has single nucleotide resolution, but requires tedious steps for sequencing and, often, cloning. By contrast, PCR-based methods are much easier to perform, although their resolution is limited. Among the PCR methods, amplicons for bisulfite-based ones are forced to be short due to the effect of bisulfite-induced DNA fragmentation, whereas those for HM-WGA-PCR are free from such concern and can be much longer. On the other hand, HM-WGA-PCR can interrogate only the regions bearing both *Hpa*II/*Hha*I and *McrBC *sites, whereas bisulfite-based PCR methods are free from such restriction. Incomplete bisulfite-conversion leads to false methylation signals in bisulfite-based methods, and incomplete digestion leads to false-positive PCR signals in HM-WGA-PCR. In anyway, HM-WGA-PCR complements bisulfite-based methods and would be a method of choice in various applications for its simplicity.

## Competing interests

The authors declare that they have no competing interests.

## Authors' contributions

YY and TI made contributions to conception, design and drafting of the manuscript. YY accepted responsibility for the acquisition, analysis and interpretation of data. Both authors read and approved the final manuscript.

## Supplementary Material

Additional file 1 **Supplemental Table S1**. The table indicates PCR conditions and primer sequences used in this study. Click here for file
